# Carpal tunnel surgery dampens thalamocortical and normalizes corticocortical functional connectivity

**DOI:** 10.1093/braincomms/fcac237

**Published:** 2022-09-22

**Authors:** Natalie R Osborne, Dimitri J Anastakis, Junseok Andrew Kim, Rima El-Sayed, Joshua C Cheng, Anton Rogachov, Kasey S Hemington, Rachael L Bosma, Camille Fauchon, Karen D Davis

**Affiliations:** Krembil Brain Institute, Krembil Research Institute, University Health Network, Toronto, M5T 1M8, Canada; Institute of Medical Science, Temerty Faculty of Medicine, University of Toronto, Toronto, M5S 1A8, Canada; Krembil Brain Institute, Krembil Research Institute, University Health Network, Toronto, M5T 1M8, Canada; Institute of Medical Science, Temerty Faculty of Medicine, University of Toronto, Toronto, M5S 1A8, Canada; Toronto Western Hospital, University Health Network, Toronto, M5T 2S8, Canada; Department of Surgery, University of Toronto, Toronto, M5T 1P5, Canada; Krembil Brain Institute, Krembil Research Institute, University Health Network, Toronto, M5T 1M8, Canada; Institute of Medical Science, Temerty Faculty of Medicine, University of Toronto, Toronto, M5S 1A8, Canada; Krembil Brain Institute, Krembil Research Institute, University Health Network, Toronto, M5T 1M8, Canada; Institute of Medical Science, Temerty Faculty of Medicine, University of Toronto, Toronto, M5S 1A8, Canada; Krembil Brain Institute, Krembil Research Institute, University Health Network, Toronto, M5T 1M8, Canada; Institute of Medical Science, Temerty Faculty of Medicine, University of Toronto, Toronto, M5S 1A8, Canada; Krembil Brain Institute, Krembil Research Institute, University Health Network, Toronto, M5T 1M8, Canada; Institute of Medical Science, Temerty Faculty of Medicine, University of Toronto, Toronto, M5S 1A8, Canada; Krembil Brain Institute, Krembil Research Institute, University Health Network, Toronto, M5T 1M8, Canada; Institute of Medical Science, Temerty Faculty of Medicine, University of Toronto, Toronto, M5S 1A8, Canada; Krembil Brain Institute, Krembil Research Institute, University Health Network, Toronto, M5T 1M8, Canada; Krembil Brain Institute, Krembil Research Institute, University Health Network, Toronto, M5T 1M8, Canada; Krembil Brain Institute, Krembil Research Institute, University Health Network, Toronto, M5T 1M8, Canada; Institute of Medical Science, Temerty Faculty of Medicine, University of Toronto, Toronto, M5S 1A8, Canada; Toronto Western Hospital, University Health Network, Toronto, M5T 2S8, Canada; Department of Surgery, University of Toronto, Toronto, M5T 1P5, Canada

**Keywords:** carpal tunnel syndrome, surgery, primary somatosensory cortex, thalamus, functional connectivity

## Abstract

Carpal tunnel syndrome is the most common entrapment neuropathy and is associated with altered brain function and structure. However, little is understood of the central mechanisms associated with its pain, symptom presentation, and treatment-related resolution. This longitudinal study evaluated carpal tunnel syndrome-related alterations in brain network communication and relationships to behavioural signs of central sensitization before and after carpal tunnel release surgery. We tested the hypothesis that carpal tunnel syndrome is associated with condition- and treatment-related plasticity in brain regions involved in somatosensation. We used quantitative sensory testing and clinical and pain questionnaires to assess sensory and pain function in 25 patients with carpal tunnel syndrome before (18 women, 7 men) and after (*n* = 16) surgery, and 25 sex- and age-matched healthy controls. We also acquired resting-state functional MRI to determine functional connectivity of two key nodes in the somatosensory system, the thalamus and primary somatosensory cortex. Seed-to-whole brain resting-state static functional connectivity analyses revealed abnormally low functional connectivity for the hand area of the primary somatosensory cortex with the contralateral somatosensory association cortex (supramarginal gyrus) before surgery (*P* < 0.01). After clinically effective surgery: (i) Primary somatosensory functional connectivity was normalized with the contralateral somatosensory association cortex and reduced with the dorsolateral prefrontal cortex (a region associated with cognitive and emotional modulation of pain) and primary visual areas (*P* < 0.001) from pre-op levels; and (ii) Functional connectivity of the thalamus with the primary somatosensory and motor cortices was attenuated from pre-op levels (*P* < 0.001) but did not correlate with temporal summation of pain (a behavioural measure of central sensitization) or clinical measures. This study is the first to reveal treatment-related neuroplasticity in resting-state functional connectivity of the somatosensory system in carpal tunnel syndrome. The findings of dysfunctional resting-state functional connectivity point to aberrant neural synchrony between the brain’s representation of the hand with regions involved in processing and integrating tactile and nociceptive stimuli and proprioception in carpal tunnel syndrome. Aberrant neural communication between the primary somatosensory hand area and the dorsolateral prefrontal cortex could reflect increased attention to pain, paraesthesia, and altered sensation in the hand. Finally, reduced thalamocortical functional connectivity after surgery may reflect central plasticity in response to the resolution of abnormal sensory signals from the periphery. Our findings support the concept of underlying brain contributions to this peripheral neuropathy, specifically aberrant thalamocortical and corticocortical communication, and point to potential central therapeutic targets to complement peripheral treatments.

## Teaser

Osborne *et al*. report dysfunctional resting-state functional connectivity within the somatosensory system of patients with carpal tunnel syndrome which normalized following carpal tunnel release surgery. Treatment-related neuroplasticity was characterized by a reduction in functional connectivity between the primary somatosensory cortex and the thalamus and prefrontal cortex.

## Graphical Abstract

**Graphical abstract:**
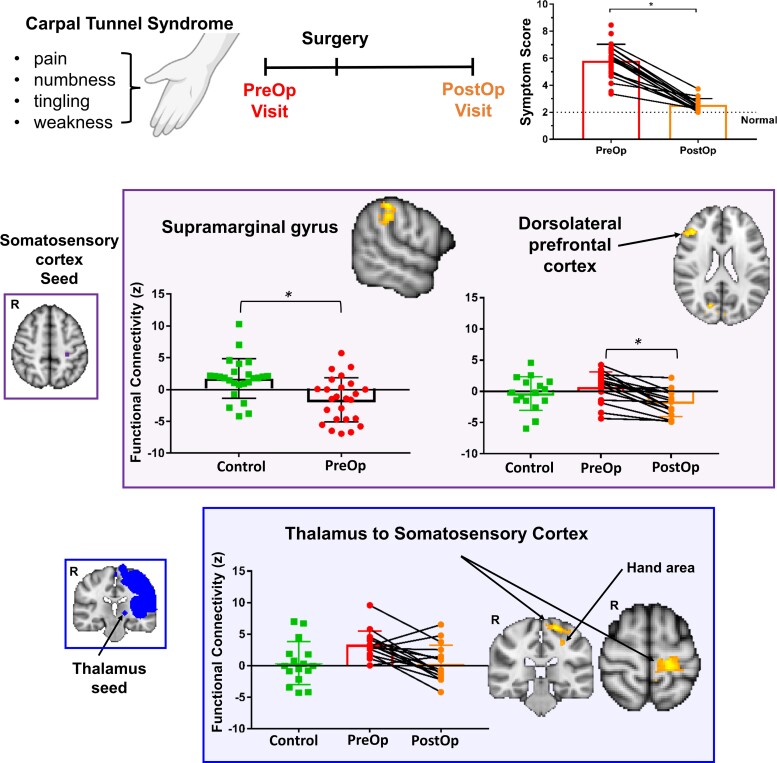

## Introduction

Carpal tunnel syndrome is the most common entrapment neuropathy, with a lifetime risk in the general population of 10%.^1^ This chronic median nerve compression is associated with persistent tingling, numbness, and pain in the hand,^2^ which can progress to loss of sensory and motor function.^3^ In addition to this peripheral aetiology, behavioural signs of central sensitization^4^ such as symptom spread outside the median nerve territory^5–7^ and proximally up the arm,^8,9^ widespread pain hypersensitivity,^10–12^ and increased temporal summation of pain (TSP)^13^ (a pain paradigm thought to reflect central sensitization) point to brain plasticity. Central sensitization has been linked to worse short-term treatment outcomes in carpal tunnel syndrome.^14^ Quantitative sensory testing (QST) has identified multiple sensory phenotypes of carpal tunnel syndrome comprising thermal or mechanical hyperalgesia and sensory loss,^15,16^ which likely reflects heterogeneity in underlying nerve pathology and corresponding neuroplasticity throughout the somatosensory system.

Aberrant neural activity from a compressed peripheral nerve to the primary somatosensory cortex (S1) is thought to drive maladaptive central neuroplasticity.^17,18^ In carpal tunnel syndrome, the S1 shows blurring of the digit representations of the affected hand^19–22^ and abnormal responses to hand stimulation,^21,23,24^ which can normalize after acupuncture^25,26^ and surgical^24,27^ treatment. Patients whose median nerve was transected and subsequently repaired exhibit abnormal responses to vibrotactile hand stimulation^28^ and tactile tasks^29^ in S1^30^ and also in the secondary somatosensory, lateral parietal, premotor, inferior temporal, and prefrontal cortices. In addition to the S1,^31–34^ it is well known that the thalamus^35–39^ is impacted by chronic pain,^40^ and thalamocortical dysrhythmia^41^ is associated with chronic neuropathic pain.^42^ Resting-state functional connectivity (rsFC) represents the synchrony of oscillatory activity between brain areas and can be an indicator of the health of a neural pathway. Characterizing rsFC changes within the somatosensory system and between it and other brain regions in carpal tunnel syndrome may help to better understand its diverse patterns of on-going sensory symptoms and their alleviation with treatments such as carpal tunnel release surgery, which reduces symptoms in 70–90% of patients.

Here we evaluated carpal tunnel syndrome abnormalities and treatment-related plasticity in the rsFC of the somatosensory system, including the thalamus and S1, to answer three main questions: How does carpal tunnel syndrome and its treatment impact (i) functional connectivity (FC) of the hand area of S1?; (ii) thalamocortical FC?; and (iii) the brain–behaviour relationship of thalamocortical FC with TSP, a reflection of central sensitization? We predicted that, due to continuous abnormal sensory signals from the hand driving enhanced spontaneous synchronization between these two regions, FC between the thalamus and S1 hand area may be stronger in patients with carpal tunnel syndrome (before surgery) compared with healthy controls (HCs) and symptom-free postoperative (post-op) patients. We also predicted that pre-operatively, patients would show behavioural signs of central sensitization such as greater TSP responses compared with HCs. To test these hypotheses, we conducted a longitudinal study that used QST and fMRI to investigate rsFC in people with carpal tunnel syndrome before and after carpal tunnel release surgery, as compared with age- and sex-matched HCs.

## Materials and methods

Additional methodological details are found in Supplementary Materials.

## Participants

This study included 50 participants: 25 patients with carpal tunnel syndrome (18 women, 7 men; mean age ± SD 49.4 ± 11.7 years) and 25 sex-matched healthy controls (HCs, 46.4 ± 9.30 years). Patients were recruited from the Toronto Western Hospital Hand Program and HCs were recruited through advertisements posted throughout hospitals of the University Health Network in Toronto, Ontario, Canada. Recruitment and data collection took place from November 2017 to October 2020. All participants provided informed, written consent to the study procedures approved by the University Health Network Research Ethics Board.

A diagnosis of carpal tunnel syndrome was based on clinical examination and in some cases also electrodiagnostic tests.^43^ Although electrodiagnostic tests were not an inclusion criteria for patient recruitment, we do note that 16 (64%) patients had electrodiagnostic studies conducted, and the results supported their clinical CTS diagnoses. We included patients between the ages of 18 to 70 years with unilateral (right hand) or bilateral carpal tunnel syndrome whose symptoms were not alleviated with conservative management (defined as three consecutive months of nightly splint wearing) and who elected to undergo carpal tunnel release surgery by Dr. Anastakis. Exclusion criteria for our study were as follows: (i) polyneuropathy/other peripheral neuropathy (e.g. double crush syndrome, cervical radiculopathy, ulnar neuropathy); (ii) atypical innervation of the hand (e.g. Martin-Gruber or Riche-Cannieu anastomoses); (iii) previous carpal tunnel release surgery; (iv) rheumatoid arthritis or degenerative osteoarthritis in the hand; (v) diabetes mellitus, renal disease or metabolic disorder; (vi) neurodegenerative disorders; (vii) other chronic pain conditions, (viii) psychiatric disorders other than depression and anxiety and (ix) pregnancy, claustrophobia and contraindications for MRI. Initial pre-screening identified 254 potentially eligible patients; subsequent screening excluded 164 patients according to the criteria mentioned above, with an additional 16 patients not interested in the study, 19 patients whose conservative management course did not finish before the study’s end date, and 28 individuals who did not attend their clinical appointments. A total of 27 patients completed the preoperative (pre-op) study visit, two of which were not included in the present analyses because they had unilateral CTS on the left hand, and 16 patients returned for the post-op visit. HCs were included in the study if they were between the ages of 18 and 70 and excluded if they had any of the nine criteria above. Additional exclusion criteria for HCs were as follows: (i) current acute pain or diagnosis of a chronic pain condition and (ii) diagnosis of depression and anxiety.

## Study protocol

The study protocol in the patient group included a pre-op assessment prior to surgical release of one or both of their carpal tunnels. HCs were assessed in a single visit. In each assessment we acquired neuroimaging, QST, and questionnaire data. Post-op assessments were also done for 16 of the patients, at least three months after surgery to allow time for the median nerve to regenerate. If a patient had surgery on both hands, the post-op visit was collected after the second surgery.

### Quantitative sensory testing

We determined mechanical, vibration, warm, cool, heat, and cold pain detection thresholds at the volar aspect of the distal phalanx (fingertip) of D2 (index finger) on the patient’s most symptomatic hand and from the right index finger in a subset of 10 HCs. We determined each participant’s ‘Pain50’ as the temperature that elicited a verbal pain rating of 50/100 (0 = no pain, 100 = worst pain imaginable).

TSP was assessed with a 30 mm × 30 mm Peltier thermode (TSA-II Neurosensory Analyzer, Medoc) set at the participant’s Pain50 temperature. A series of 10 consecutive 1 s thermal stimuli were applied manually at 0.33 Hz to the right volar forearm (15 cm from the wrist). Participants verbally rated the pain (0–100) after each stimulus. A practice run was done before the series used to determine TSP. The TSP effect was calculated as the percentage change in pain from the first stimulus to the peak pain rating within the series as done previously.^44^ Five patients and five HCs were excluded from the TSP analyses because they did not have pain evoked by the first three stimuli.

### Questionnaires

Participants self-identified their sex in a socio-demographic questionnaire with the binary options of male and female. The severity of carpal tunnel syndrome was assessed with the patient-completed Boston Carpal Tunnel Questionnaire (BCTQ), a validated tool^45,46^ that contains two subscales. The symptom severity scale contains 11 questions about the occurrence and duration of sensations such as pain, numbness, and tingling. Each item is rated one-five, with one representing a ‘normal’ response (no symptoms) and five indicating ‘very serious’ or constant symptoms. The numerical responses to each question are summed, and the total divided by the number of questions (11) to calculate the symptom score. The functional status scale consists of eight questions that assess hand function. Each item is rated 1–5 and the numerical responses are summed and divided by eight to obtain the functional score. Because a ‘normal’ response is indicated by a one, a symptom-free hand would have a symptom severity score of one (1 × 11/11) and a functional status score of one (1 × 8/8), and therefore a total BCTQ score of two. The most severe BCTQ score possible is 10. Here we report the BCTQ for the right hand only. Symptom distribution was delineated with the Katz Hand Diagram,^47^ assessed (by N.R.O.), and classified as indicating either a ‘median nerve’ or ‘extra-median nerve’ symptom distribution. An extra-median distribution could include areas innervated by the radial or ulnar nerve (e.g. little finger) or ‘whole hand’ i.e. ‘glove distribution’.^7^

We used two questionnaires to assess chronic pain: The painDETECT questionnaire^48^ was used to measure the likelihood of the presence of neuropathic pain. The Brief Pain Inventory^49^ (BPI) was used to assess pain severity at present, average pain, and pain at its worst and least in the past 24 h.^50^ The BPI assesses pain from 0 (no pain) to 10 (pain as bad as you can imagine). Here we report the average of these four ratings as patient’s ‘average pain severity’.

### Neuroimaging acquisition

Neuroimaging data were acquired with a 3 T GE Signa MRI scanner fitted with an 8-channel phase-array head coil (GE Medical Systems, Chicago, IL). The neuroimaging acquisition protocol included (i) a high resolution T_1_-weighted anatomical scan [3D IR-FSPGR sequence; 180 axial slices; repetition time (TR), 7.8 ms; echo time (TE), 3 ms; flip angle, 15°; 256 × 256 matrix; 1 mm × 1 mm × 1 mm voxels), and (ii) a T_2_*-weighted eyes-closed resting-state fMRI scan lasting 9 min and 14 s (echo-planar imaging sequence; 36 slices; TR, 2000 ms; TE, 30 ms; 64 × 64 matrix; 3.125 mm × 3.125 mm × 3.125 mm voxels).

## Behavioural and questionnaire data analysis

GraphPad Prism 7 was used for statistical analyses of the QST and questionnaire data and correlations with neuroimaging metrics. Two-sample unpaired *t*-tests or Mann–Whitney U tests were used for group comparisons and paired one- and two-tailed *t*-tests or Wilcoxon rank sum tests were used for pre/post-op comparisons. Pearson or Spearman’s correlations were used as appropriate. Sensory threshold data from a subset of HCs (*n* = 10) was used for descriptive statistical purposes to assess pre-op sensory gain and loss.

## Pre-processing of functional MRI data

The fMRI Expert Analysis Tool (FEAT) in FMRIB Software Library (FSL) version 5.0.1 was used to pre-process the data. We removed the first four volumes of the data from the resting-state scan. We used FEAT’s Brain Extraction Tool (BET) to remove data arising from non-brain voxels from the functional data and performed motion correction using Motion Correction FMRIB’s Linear Image Registration Tool (MCFLIRT).^51^ We then inspected participants’ absolute and relative root mean square displacements (RMSs), generated by running MCFLIRT on the functional data, to ensure they did not exceed the cut-off of 2.5 mm (absolute RMS) and 0.30 mm (relative RMS). T_1_-weighted anatomical images were skull-stripped with OptiBET.^52^ FMRIB’s Linear Image Registration Tool (FLIRT)^51^ (rigid body transformation with 6 degrees of freedom) was used to register each participant’s functional images to their skull-stripped T_1_-weighted anatomical image, followed by non-linear registration to MNI152–2 mm space with FMRIB’s Non-linear Image Registration Tool. We used aCompCor,^53^ as described previously,^54^ to further remove physiological and scanner-related noise. aCompCor was used to identify the top five white matter and cerebrospinal fluid components and six motion parameters, and then to regress these out of the functional data. Spatial smoothing was applied using a 4 mm FWHM Gaussian spatial smoothing kernel. Finally, we temporally filtered the data using a high-pass filter (0.01 Hz cut-off) to remove scanner-related signal drift and a low-pass filter (0.1 Hz cut-off) to reduce physiological noise.

## Functional connectivity analyses

We used three approaches to examine FC abnormalities associated with carpal tunnel syndrome and its treatment: The first set of analyses determined the static FC of the hand area of S1 with the whole brain (i.e. seed-to-whole brain). The second set of analyses determined the static FC of the thalamus with other somatosensory regions (i.e. seed-to-mask, seed-to-seed). The third analysis determined the brain–behaviour relationships between FC and behavioural and clinical measures, such as that between thalamus-S1 FC and TSP. A cluster-based statistical threshold of Z > 2.3 and *P* < 0.05 with a Family Wise Error (FWE) correction was used for all FC analyses to correct for multiple comparisons. We also performed dynamic and sex-disaggregated FC analyses, which can be found in Supplementary material.

### Definition of seeds for resting-state FC

The hand is the primary location of symptoms experienced in carpal tunnel syndrome. Therefore, we placed a 3 mm radius spherical seed in the hand area of the left S1 to explore potential disease and treatment-related alterations in FC. The S1 seed location (MNI: −28, −30, 50) was previously published based on fMRI activity in response to painful stimulation of the hand in healthy adults.^55^ We used a spherical seed in the left sensory thalamus (4 mm radius, MNI: −16, −24, 0) that was previously published in our lab.^44^ These left hemisphere seeds were located contralateral to the right hand because all patients had carpal tunnel syndrome affecting the right median nerve, although many patients additionally had left hand symptoms.

### Analysis 1a: determination of abnormal S1 FC with the whole brain

In analysis 1a, we assessed whether left S1-to-whole brain FC was abnormal in carpal tunnel syndrome patients compared to age- and sex-matched HCs. First, we transformed the left S1 hand area seed non-linearly from standard MNI space into native space for each participant. Next, we extracted each participant’s average timeseries of the S1 seed from their rs-fMRI data and used it in a first-level GLM analysis in FSL’s FEAT, where the time series of the S1 seed was entered as a regressor to identify voxels throughout the whole brain that were significantly functionally connected to the seed. The output from each participants’ first-level analysis was then entered into a second-level group analysis using FEAT’s two-group difference model and FLAME 1 + 2. This model uses a two-sample unpaired *t*-test to compare S1-to-whole brain FC in the patient and HC groups.

### Analysis 1b: determination of treatment-related plasticity of the S1 FC with the whole brain

We ran a first-level GLM analysis to examine seed-to-whole brain FC of the same S1 hand area seed (with the method described in Analysis 1a) using the post-op rs-fMRI data acquired from 16 patients (12 women, 4 men). To examine treatment-related plasticity, we performed a paired two-group difference (i.e. two-sample paired *t*-test) analysis in FEAT comparing pre- to post-op S1-to-whole brain FC.

### Analysis 1c: exploration of S1 FC abnormalities post-treatment

To investigate whether the abnormal S1 FC that we found in Analysis 1a normalized after treatment, we conducted an exploratory analysis to compare S1-to-whole brain static FC in patients post-op (*n* = 16) with sex- and age-matched HCs (*n* = 16). The first-level S1-to-whole brain FC analyses that were generated from Analyses 1a and b were entered into a second-level group analysis using FEAT’s two-group difference model and FLAME 1 + 2.

### Analysis 2a: determination of abnormal thalamocortical FC

A mask of somatosensory areas was used to examine abnormal thalamocortical FC in carpal tunnel syndrome. We used a similar approach as our seed-to-whole brain first-level FC analysis for each participant as described in Analysis 1a, this time using the seed located in the left sensory thalamus. We created a mask of the left somatosensory regions using FSL’s Juelich atlas that consisted of the left insula and primary and secondary somatosensory cortices and applied it to a second-level analysis (again using FEAT’s two-group difference model and FLAME 1 + 2) to compare FC between the thalamus and somatosensory mask in pre-op patients versus HCs.

### Analysis 2b: determination of treatment-related plasticity of thalamocortical FC

To examine treatment-related plasticity of the FC between the thalamus and other somatosensory areas, we used a similar first-level thalamus-to-whole brain FC analysis as described in Analysis 2a. For this analysis, we used the pre- and post-op resting-state data, and the same left somatosensory mask was applied to a second-level paired two-group difference analysis in FEAT. We also conducted a second-level two-group difference analysis comparing thalamic FC with the somatosensory mask in post-op patients and HCs.

### Analysis 3: brain–behaviour relationships between FC and pain

To examine the relationship between an individual’s TSP and their thalamocortical FC, we ran a seed-to-seed analysis. First, we extracted the average timecourse of the thalamus seed for each participant using the fslmeants command. We repeated this process for a 3 mm radius spherical S1 seed located in Broadman Area 3a (BA3a), MNI: −34, −22, 42. This seed was selected because strong FC between this region and the thalamus has been linked with TSP in healthy adults.^44^ FC between the thalamus and S1 BA3a seeds was calculated using MATLAB R2016b for each participant, and then compared between pre-op patients and HCs with a two-sample *t*-test. Spearman’s correlations were performed to compare patients’ and HCs’ thalamus-S1 BA3a FC values with their TSP responses.

We also explored whether any significant differences in FC that were found between patients and HCs or within patients pre and post surgery were correlated with measures of patients’ clinical and pain severity (BCTQ symptom score, average pain (BPI), and painDETECT scores). To do this, we entered pre-op patients’ first-level S1 and thalamus FC analyses into second-level regression analyses in FEAT (single-group average with additional covariate), with the statistically significant clusters from the group difference used as a mask, and the demeaned pre-op clinical scores entered as a covariate.

## Statistical analysis

The normality of the QST and questionnaire data was assessed with the D’agostino & Pearson and the Shapiro-Wilk normality tests using GraphPad Prism 7.0. Experimenter blinding to patients’ pre- or post-op state was not possible because carpal tunnel release surgery produces a visible scar on the wrist. We did not perform a power analysis for the current study due to the challenges in accurately calculating effect sizes and power for rsFC studies. Our current study’s chronic pain patient sample size is similar to other chronic pain imaging studies and reflects the greater challenge in recruiting participants in this disease group compared to healthy controls.

### Data availability

Data pertaining to the results of this study may be obtained through the corresponding author upon reasonable request.

## Results

### Descriptive statistics and clinical characteristics of patients with carpal tunnel syndrome

**Figure 1:**
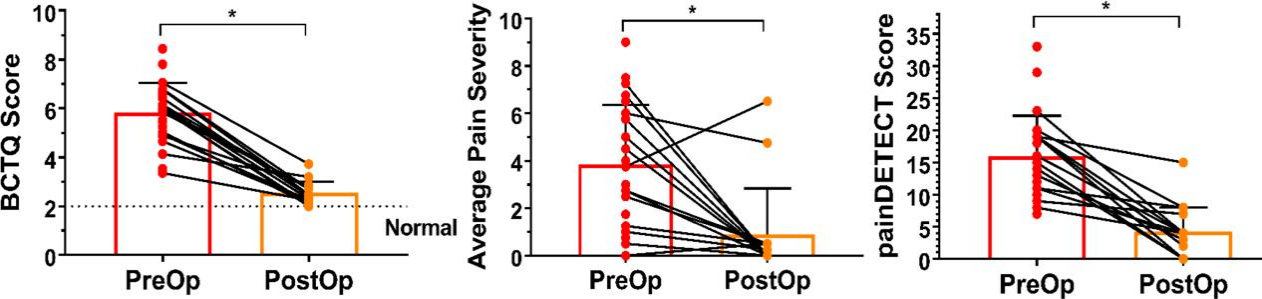
**Clinical characteristics pre- and post-carpal tunnel release surgery.** CTS sensory symptoms and functional impairment were captured by patients’ BCTQ scores (2 = normal, 10 = most severe). The group average BCTQ score significantly improved from 5.9 ± 1.2 (group mean ± SD) pre-op (*n* = 24) to 2.5 ± 0.4 post-op (*n* = 16) (*P* < 0.0001, unpaired one-tailed *t*-test). Average pain severity represents the mean of the scores from the BPI for best and worst pain in the past 24 h, pain at present, and average pain (0 = no pain, 10 = pain as bad as you can imagine). Average pain severity was significantly reduced from 3.9 ± 2.5 (pre-op, *n* = 23) to 0.9 ± 1.9 (post-op, *n* = 15) following surgical treatment (*P* < 0.001, Mann–Whitney one-tailed test). Neuropathic pain scores (as measured by painDETECT) were also significantly reduced after surgery (16.0 ± 6.2 pre-op, 4.2 ± 3.8 post-op, *P* < 0.001, Mann–Whitney one-tailed test). * indicates *P* < 0.05, error bars represent SD. BCTQ = Boston carpal tunnel questionnaire; CTS = carpal tunnel syndrome, Pre-op = preoperative, post-op = postoperative.

Clinical and demographic information are presented in Table 1 and Fig. 1 as well as Supplementary Table 1 and Supplementary material. There were no significant differences in age between patients and HCs. Pre-operatively, nine patients had PainDETECT scores below 13 (indicating a low likelihood of a neuropathic pain component), six had scores of 13–18 (indicating their pain may have a neuropathic component), and 10 had scores above 19 (indicating a high likelihood of neuropathic pain). Based on the Katz Hand Diagram, 17 patients had a median distribution of symptoms (i.e. consistent with the median nerve’s innervation territory), whereas seven showed an extra-median symptom distribution (symptoms beyond the median nerve territory).

**Table 1:** Demographic and clinical characteristics

	CTS pre-op	CTS post-op	HCs
Total *n*	25	16	25
W/M	18/7	12/4	18/7
Age (years)	49.4 ± 11.7	52.1 ± 12.0	46.4 ± 9.3
BCTQ (score: 2–10)	5.9 ± 1.2	2.5 ± 0.4*	
painDETECT (score: 0–38)	16.0 ± 6.2	4.2 ± 3.8*	
BPI Average Pain Severity (score: 1–10)^a^	3.9 ± 2.5	0.9 ± 1.9*	

Group data displayed as mean ± standard deviation.

* *P* < 0.05. Paired t-tests compared the subset of CTS patients with pre versus post-op data.

BCTQ, Boston Carpal Tunnel Questionnaire; BPI, Brief Pain Inventory; HCs, healthy controls; W; Women; M, Men.

^a^ Average Pain Severity score was missing from one post-op CTS patient.

All three indicators of pain and pain-related impact were improved after surgery (see Table 1 and Fig. 1, group mean ± SD reported). The mean painDETECT scores fell significantly [16.0 ± 6.2 pre-op (*n* = 25) to 4.2 ± 3.8 post-op (*n* = 16); *P* < 0.001, Mann–Whitney one-tailed test] as did the pain ratings [3.9 ± 2.5 pre-op (*n* = 23) to 0.9 ± 1.9 post-op (*n* = 15); *P* < 0.001, Mann–Whitney one-tailed test]. The BCTQ scores also improved post-operatively [pre-op (*n* = 24): 5.9 ± 1.2, post-op (*n* = 16): 2.5 ± 0.4, unpaired one-tailed *t*-test, *P* < 0.0001].

### Less temporal summation of pain and a mix of sensory gain and loss in carpal tunnel syndrome

**Figure 2:**
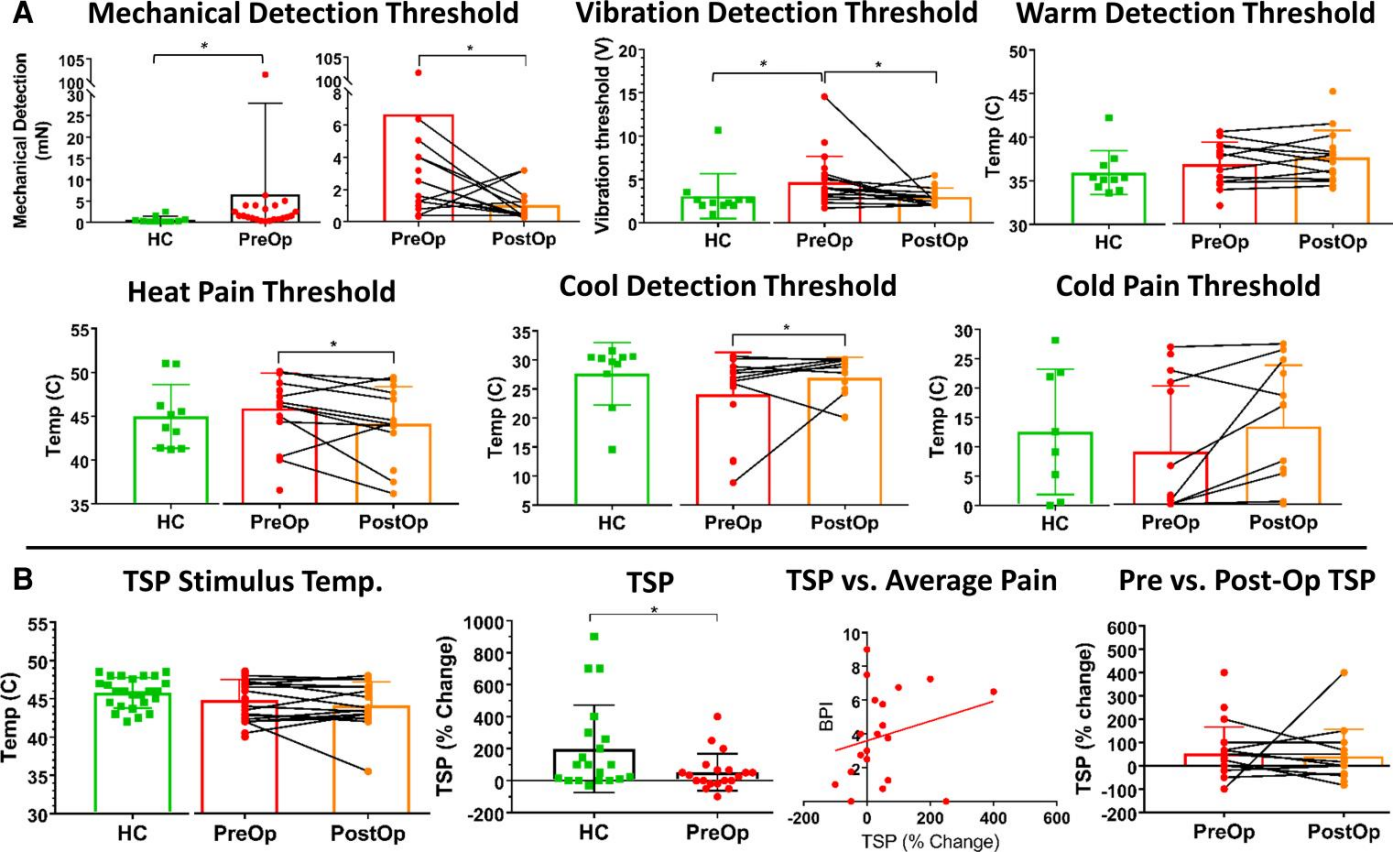
**Quantitative sensory testing in patients and HCs.** (**A**) Mechanical, vibration and thermal thresholds on the most affected digit (D2) on the most affected hand in patients with carpal tunnel syndrome before and after surgery, as well as a group of 10 HCs. Statistical comparisons between preoperative patients and HCs are for descriptive purposes only. Patients with carpal tunnel syndrome’s mechanical and vibration thresholds were significantly higher than HCs before surgery (MDT *P* < 0.001; VDT *P* < 0.01, Mann–Whitney one-tailed tests), but then decreased after surgery compared to pre-op levels (MDT *P* = 0.03; VDT *P* = 0.01, Wilcoxon matched-pairs signed-rank tests, one-tailed). Pre-op thermal thresholds did not differ from HCs (WDT & CDT Mann–Whitney two-tailed tests; HPT two-tailed unpaired *t*-test; CPT Welch’s two-tailed test). Heat and cold pain thresholds did significantly change compared to pre-op levels following surgery (HPT *P* = 0.0488; CPT *P* = 0.0414, one-tailed paired t-tests), while warm and cool detection thresholds did not (WDT: one-tailed paired *t*-test, CDT: Wilcoxon matched-pairs signed-rank test, one-tailed). (**B**) There were no significant differences in the temperature required to elicit a pain rating of 50 out of 100 (Pain50) among pre and post-op patients and HCs (Mann–Whitney two-tailed tests). TSP responses in pre-op patients were significantly lower than HCs (*P* = 0.048, Mann–Whitney two-tailed test) and did not correlate with patients’ average pre-op pain scores (BPI) (Spearman’s correlation). After surgery, patient’s TSP responses decreased from pre-op averages but not significantly (Wilcoxon matched-pairs signed-rank test, two-tailed). * indicates *P* < 0.05. Thermal thresholds could not be collected for some patients due to equipment malfunction or time constraints. Error bars represent SD. Pre-op = preoperative, Post-op = postoperative, HC = healthy control, TSP = temporal summation of pain, BPI = Brief Pain Inventory.

The somatosensory thresholds in the HCs and the most affected digit (D2, index finger) of the most affected hand in the carpal tunnel syndrome group before and after surgery are shown in Fig. 2. Here, we report the group mean ± SD for all sensory thresholds. A comparison of the subset of HCs with sensory threshold data from D2 (*n* = 10) to the patients’ pre-op state (*n* = 22) showed that patients had significantly (Mann–Whitney one-tailed test, *P* = 0.0005) higher mechanical detection thresholds (6.6 ± 21.3 mN) than the HCs (0.69 ± 0.7 mN). Pre-operatively, patients (*n* = 20) also had significantly higher vibration detection thresholds than HCs (*n* = 11) (pre-op mean 4.7 ± 3.0 V, HC mean 3.1 ± 2.6 V; Mann–Whitney one-tailed test, *P* = 0.0027). However, these patients did not differ pre-operatively from HCs in terms of their group average warm detection thresholds (patients (*n* = 15): 36.8 ± 2.5°C; HCs (*n* = 10): 35.93 ± 2.5°C, Mann–Whitney two-tailed test, *P* = 0.30), cool detection thresholds (patients (*n* = 14): 24.1 ± 7.3°C; HC (*n* = 10): 27.6 ± 5.4°C, Mann–Whitney two-tailed test, *P* = 0.07), heat pain thresholds [patients (*n* = 15): 45.7 ± 4.0°C; HCs (*n* = 10): 45.0 ± 3.6°C, two-tailed unpaired *t*-test, *P* = 0.63], or cold pain thresholds [patients (*n* = 14): 9.0 ± 11.2°C; HCs (*n* = 8): 12.5 ± 10.7°C, Welch’s two-tailed *t*-test, *P* = 0.47].

After surgery, patients’ mechanical and vibration detection thresholds were significantly lower compared to their pre-op levels (mechanical detection: Wilcoxon matched-pairs signed-rank test, one-tailed, *P* = 0.032) from a geometric mean of 6.6 ± 21.2 mN pre-op (*n* = 22) to 1.0 ± 1.0 mN post-op (*n* = 14); (vibration detection: Wilcoxon matched-pairs signed-rank test, one-tailed, *P* = 0.0148) from 4.7 ± 3.0 V (pre-op, *n* = 20) to 3.0 ± 1.1 V (post-op, *n* = 14). Heat pain thresholds also decreased significantly (one-tailed paired *t*-test, *P* = 0.0488) from 45.8 ± 4.0°C pre-op (*n* = 15) to 44.1 ± 4.4°C post-op (*n* = 14), and cold pain thresholds increased significantly (one-tailed paired *t*-test, *P* = 0.0414) from 9.0 ± 11.2°C pre-op (*n* = 14) to 13.3 ± 10.5°C post-op (*n* = 13). However, the warm thresholds (pre-op (*n* = 15): 36.8 ± 2.5°C, post-op (*n* = 14): 37.6 ± 3.1°C, one-tailed paired *t*-test, *P* = 0.4849) and cool thresholds (pre-op (*n* = 14): 24.1 ± 7.3, post-op (*n* = 13): 26.9 ± 3.6, Wilcoxon matched-pairs signed-rank test, one-tailed, *P* = 0.2129) were not significantly different before and after surgery.

There was no significant difference (Mann–Whitney two-tailed test, *P* = 0.264) in the average temperature that elicited a pain rating of 50 in pre-op patients (44.9 ± 2.7°C) compared with HCs (45.8 ± 1.9°C), nor in post-op average pain50 temperatures (44.1 ± 3.1°C) compared with HCs (Mann–Whitney two-tailed test, *P* = 0.065). Prior to surgery, the patients exhibited significantly less TSP compared with HCs (53% ± 115 in carpal tunnel syndrome [*n* = 20), 199% ± 273 in HCs (*n* = 20), Mann–Whitney two-tailed test, *P* = 0.048]. Patient’s pre-op TSP responses were not correlated (Spearman *r* = 0.27, two-tailed *P* = 0.25) with their average pain scores (BPI). After surgery, the average TSP response (43% ± 114, *n* = 16) decreased from the pre-op average, but not significantly (Wilcoxon matched-pairs signed-rank test, two-tailed, *P* = 0.65).

## Functional connectivity findings

### Abnormally low S1 FC with the somatosensory association cortex in carpal tunnel syndrome

**Figure 3:**
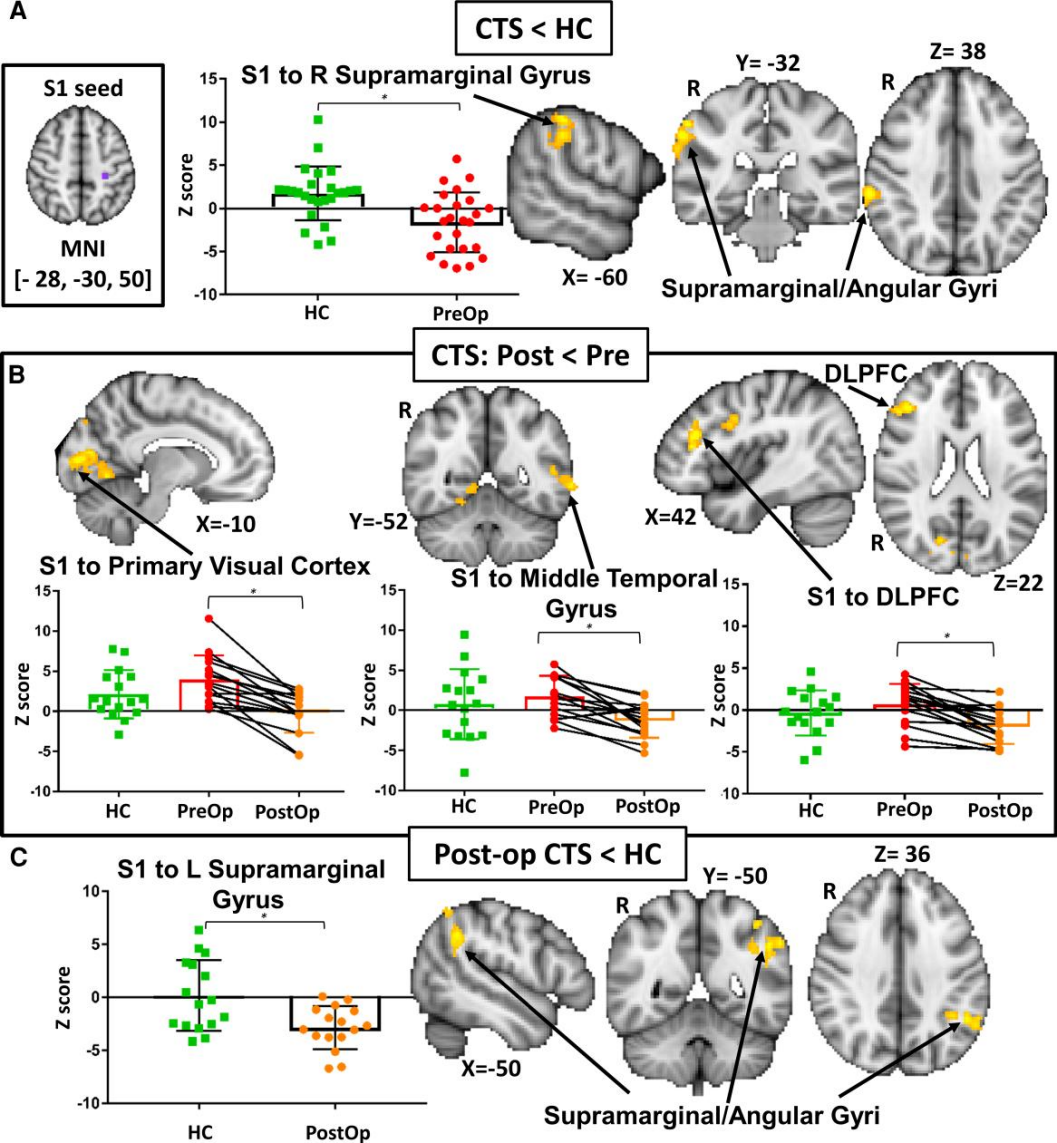
**S1 hand-to-whole brain FC in patients and HCs.** (**A**) A left S1 hand area seed-to-voxel whole brain FC analysis compared pre-op CTS patients (*n* = 25) to HCs (*n* = 25) (Analysis 1a). CTS patients had abnormally low left S1 FC with the right somatosensory association cortex (BA 40, supramarginal and angular gyri). (**B**) The left S1 seed-to-whole brain FC comparison within patients with CTS before and after treatment (Analysis 1b, *n* = 16) revealed that post-op, patients had attenuated S1 FC with the primary visual cortex (BA 17, intracalcarine cortex), middle and superior temporal gyri (BA 21 & 22), and DLPFC (BA 9 & 46, middle and inferior frontal gyri). (**C**) Analysis 1c comparing S1 seed-to-whole brain FC between post-op patients and HCs (*n* = 32) revealed normalization of left S1-right somatosensory association cortex FC, but abnormally low S1 FC with the left somatosensory association cortex (supramarginal & angular gyri) in post-op patients. Graphs represent the averaged z-scores from participants in each group (horizontal lines represent the mean and the vertical lines represent the SD) extracted from a 2 mm spherical seed centred on the peak coordinates of one of the significant clusters from the contrast. * indicates *P* < 0.05 (FWE-corrected for multiple comparisons). S1 = primary somatosensory cortex, FC = functional connectivity, CTS = carpal tunnel syndrome, HC = healthy controls, BA = Broadman area, DLPFC = dorsolateral prefrontal cortex, R = Right, Post = Post-op, Pre = Pre-op.

In our neuroimaging analysis 1a, we found that compared with HCs, the pre-op carpal tunnel syndrome group (*n* = 25) had lower FC between the left S1 hand area seed and a cluster that included the right somatosensory association cortex, specifically the supramarginal gyrus (Broadman Area (BA) 40) and angular gyrus (*P* < 0.01; see Fig. 3 and Table 2).

**Table 2:** Main functional connectivity findings

Analysis 1a. S1 hand area-to-whole brain FC, all CTS (*n* = 25) versus HC (*n* = 25)						
Contrast	*Z*	*P*	MNI Coordinates			Brain Regions
			x	y	z	
CTS < HC	3.99	0.00732	62	−32	38	BA 40, Somatosensory Association Cortex Inferior Parietal Lobule (Supramarginal and Angular Gyri)
**Analysis 1b. S1 hand area-to-whole brain FC, Pre versus Post-op CTS (*n* = 16)**						
Post < Pre	4.64	2.96e-12	−10	−86	8	BA 17, Bilateral Primary Visual Area (Intracalcarine Cortex)
	4.84	0.0013	42	32	22	BA 9 & 46, Dorsolateral Prefrontal Cortex (Middle Frontal Gyrus, Inferior Frontal Gyrus)
	3.48	0.0253	−66	−52	0	BA 21 & 22, Middle & Superior Temporal Gyrus BA 39, Angular Gyrus
**Analysis 1c. S1 hand area-to-whole brain FC, Post-op CTS versus HC (*n* = 32)**						
CTS < HC	3.46	0.000425	−24	−74	60	Lateral Occipital Cortex BA 40, Somatosensory Association Cortex Inferior Parietal Lobule (Supramarginal & Angular Gyri)
**Analysis 2b. Thalamocortical FC, Pre versus Post-Op CTS (*n* = 16)**						
Post < Pre	4.17	0.000523	−18	−30	64	BA 1, Primary Sensory Cortex BA 4, Primary Motor Cortex BA 5, Sensory Association Cortex BA 7, Visual Motor Area (Pre and Postcentral gyri)

Peak MNI coordinates, *Z*, and *P* values are reported for significant clusters. Analysis 1a shows significant clusters found in the S1 (hand area) seed-to-voxel whole brain functional connectivity (FC) analyses comparing pre-op carpal tunnel syndrome (CTS) patients (*n* = 25) and healthy controls (HCs) (*n* = 25), Analysis 1b shows significant clusters in the S1 hand area seed-to-voxel whole brain FC analysis comparing CTS pre versus post-op (*n* = 16), with Analysis 1c comparing CTS post-op versus HCs (*n* = 32). Analysis 2b shows significant clusters from the thalamus seed to somatosensory system mask FC analysis comparing pre versus post-op CTS patients (*n* = 16). Brain regions are provided using FSL’s Harvard-Oxford Cortical Atlas & Talairach Daemon Label tools. Thresholded at *P* < 0.05 (FWE-corrected for multiple comparisons).

### S1 hand area FC with the prefrontal and visual cortices decreases after carpal tunnel surgery

In Analysis 1b, we examined treatment effects on FC by comparing data obtained from patients with carpal tunnel syndrome before and after surgery (*n* = 16). We found that FC between the S1 hand area and the prefrontal cortex was attenuated after surgery, specifically in BA 9 and 46, corresponding to the right dorsolateral prefrontal cortex (DLPFC, *P* = 0.001). Post-op patients also had decreased S1 FC with bilateral primary visual cortices (BA 17, *P* < 0.001), and a cluster (*P* = 0.02) including the left middle and superior temporal gyri (BA 21 & 22) and angular gyrus (BA 39) (see Fig. 3 and Table 2 for cluster coordinates).

### Persistent post-treatment abnormalities in S1 FC with the somatosensory association cortex

In analysis 1c, we examined whether the abnormally low FC between the left S1 and the right somatosensory association cortex became normalized in patients following treatment. We found no differences in left S1 FC with the right somatosensory association cortex between post-op patients and HCs. However, post-operatively, patients did have abnormally low left S1 FC with a cluster in the left somatosensory association cortex, including the supramarginal and angular gyri, as well as the lateral occipital cortex (Fig. 3 and Table 2).

### FC between the thalamus and S1 decreased following surgery

**Figure 4:**
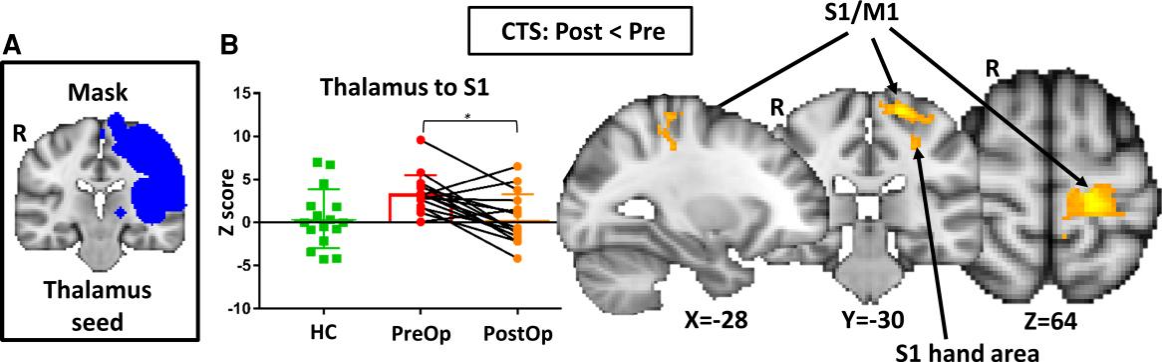
**Thalamocortical FC in patients.** (**A**) We calculated FC between a seed in the left thalamus and a mask of the somatosensory system, which included left S1, S2, and insula. (**B**) Compared to pre-op patients, post-op patients had attenuated FC between the left thalamus and a cluster including the left S1 (postcentral gyrus, BA 1), M1 (precentral gyrus, BA 4) and visual motor (BA 7) and sensory association (BA 5) areas (Analysis 2b, *n* = 16). Graphs represent the averaged z-scores from participants in each group (horizontal lines represent the mean and the vertical lines represent the SD) extracted from a 2 mm spherical seed centred on the peak coordinates of one of the significant clusters from the contrast. * indicates *P* < 0.05, (FWE-corrected for multiple comparisons). S1 = primary somatosensory cortex, M1 = primary motor cortex, FC = functional connectivity, CTS = carpal tunnel syndrome, HCs = healthy controls, BA = Broadman area, R = right, Post-op = postoperative, Pre-op = preoperative.

Analysis 2a did not reveal any significant differences in FC between the thalamus and somatosensory mask in patients with carpal tunnel syndrome before surgery compared to HCs. However, Analysis 2b revealed that post-operatively, patients had decreased FC between the thalamus and a cluster including the left S1 (BA 1), primary motor cortex (BA 4), sensory association cortex (BA5) and visual motor area (BA 7) compared to patients pre-operatively (*P* < 0.0001). There were no differences in thalamic FC with the somatosensory mask between post-op patients and HCs (Fig. 4 and Table 2).

### No differences in thalamic-S1 FC between patients and HCs

The seed-to-seed analyses of FC between the thalamus and S1 BA3a did not reveal any significant differences between patients pre-operatively (*n* = 25) and HCs (Mann–Whitney two-tailed test, *P* = 0.6862) or post-operatively (*n* = 16) and HCs (Mann–Whitney two-tailed test, *P* = 0.30). See Supplementary Figure 1.

### FC is not associated with TSP or clinical pain

Analysis 3 investigated brain–behaviour relationships between FC and behavioural and clinical pain measures. We found no significant correlations between thalamus and S1 BA3a FC with the TSP scores (*n* = 22) in patients *(*Spearman’s *rho*=−0.18, *P* = 0.41) or in HCs (Spearman’s *rho* = 0.10, *P* = 0.66, see Supplementary Figure 1). The regression analyses in FEAT did not reveal any significant relationships between pre-op BCTQ symptom, average pain, or painDETECT scores and regions that showed abnormalities or plasticity in their FC with the S1 and thalamus seeds (see Supplementary Figure 2).

## Discussion

This is the first study to identify treatment-related brain plasticity in rsFC within the somatosensory system in carpal tunnel syndrome. We demonstrated that these patients exhibit altered communication at rest between the S1 and other brain regions. Our main findings are as follows: (i) Before surgery, patients have abnormally low rsFC between the hand area of S1 and the contralateral somatosensory association cortex, specifically in regions associated with pain and discerning somatosensory stimuli. Then, FC of S1 (corresponding to the operated hand) with the contralateral somatosensory association cortex normalized after surgery that relieved pain and somatosensory symptoms, but S1 FC with the ipsilateral somatosensory cortex was abnormally low. (ii) After surgery, the S1 FC was attenuated from pre-op levels with the DLPFC, a region associated with attentional and emotional modulation of pain, as well as with the primary visual cortex. (iii) FC between the thalamus and S1 (as well as M1) was also attenuated after surgery. These findings suggest that thalamocortical FC reflects the presence (and subsequent reduction) of pain, numbness, and paraesthesia in carpal tunnel syndrome, and supports the proposition that persistent abnormal peripheral input from the hand can drive functional changes throughout the somatosensory system^7,17^ Our findings of brain plasticity in carpal tunnel syndrome support the importance of considering CNS contributions to symptoms when diagnosing and treating this neuropathy.^17,56^

There are several proposed mechanisms of S1 plasticity in carpal tunnel syndrome. Hebbian neuroplasticity driven by abnormally enhanced temporal synchrony of signals from paraesthesias emanating from the fingers may cause S1’s digit maps to blur,^21^ and larger and slower S1 responses to stimulation may reflect increased afferent drive from the hand or coactivation of neighbouring digits’ cortical sites.^23^ A study of S1 rs-fMRI activity reported increased FC with the ipsilateral, and decreased FC with the contralateral, hemisphere in patients compared to HCs, including lower S1 FC with the contralateral supramarginal gyrus.^57^ They also found decreased amplitude of low frequency fluctuation (ALFF) in S1, which they proposed reflected lower local activity that led to desynchronization with the contralateral S1 and subsequent cortical disinhibition^19^ that facilitated the ipsilateral FC.^57^

Our study also identified abnormally low static rsFC between the left S1 and the right somatosensory association cortex (supramarginal and angular gyri) in carpal tunnel syndrome patients before surgery. Bilateral tactile hand stimulation has been shown to activate the right supramarginal gyrus,^58^ and there is right-lateralized processing of bilateral painful and non-painful stimuli in BA40.^59^ As part of the somatosensory association cortex, the supramarginal gyrus is thought to be involved in integrating visual and tactile stimuli,^60^ as well as proprioception,^61^ and stroke-induced damage to this area has been linked with proprioceptive difficulties^62,63^ and tactile agnosia.^64^ Lesions to the left angular gyrus can lead to ‘finger agnosia’, which is defined as a loss of the ability to distinguish, name, recognize, and even selectively move fingers.^65^ Transcranial magnetic stimulation to the angular and supramarginal gyri can temporarily induce finger agnosia in healthy adults.^66^ Therefore, decreased left hand area S1 rsFC to the right somatosensory association cortex could reflect a patient’s reduced tactile acuity and sensitivity, and functional difficulties associated with their hand. Abnormally low brain activation in BA40 in response to vibrotactile hand stimulation has been shown in patients whose median nerves were transected and subsequently repaired and may be linked to incomplete nerve regeneration and persistent impairments in mechanical and vibration detection.^28^ Interestingly, although left S1 FC with the right somatosensory association cortex normalized in patients after surgery, they showed abnormally low left S1 FC with the left supramarginal and angular gyri compared with HCs. It is unclear what is driving persistent abnormalities between the left S1 hand area and its ipsilateral somatosensory association cortex in patients whose right hand symptoms had resolved, although it is possible mild symptoms in the untreated left hand could contribute to aberrant bilateral processing in somatosensory association areas.

One of our key findings was the reduction of S1 rsFC with the DLPFC following surgery. The DLPFC is associated with attentional modulation of tactile processing and memory,^67,68^ and top-down cognitive engagement of descending pain modulation, as well as reducing the emotional unpleasantness of pain. Alterations in DLPFC structure and function, including abnormally strong FC with the S1,^33^ are reported in chronic pain.^69,70^ Non-invasive brain stimulation of the DLPFC can reduce somatosensory cortical excitability in sustained pain,^71^ and a dynamic causal model of pain processing found an additional forward and backward connection between S1 and right DLPFC in chronic pain but not HCs, which they proposed could reflect increased attention to and catastrophizing of pain.^72^ Although we did not find any correlation of the S1-DLPFC FC with symptom or pain scores pre-operatively, it is possible that such a relationship may exist with other individual factors such as pain catastrophizing. Therefore, it is possible that enhanced S1-DLPFC FC before surgery reflects greater attention to abnormal sensory signals from the hand.

Another key finding in our study is that of decreased thalamocortical FC post-operatively involving S1 and M1 compared to pre-op levels. We suggest that thalamic-S1 FC reflects and is maintained by on-going aberrant input from the median nerve. We propose its reduction post-operatively occurs because this afferent input is attenuated, as signified by the symptom relief following surgery. Alternately, stronger rsFC could reflect cortical disinhibition, a compensatory mechanism to magnify weaker sensory input from a damaged nerve to maintain sensory perception in the hand,^22^ as proposed to occur in patients whose median nerves were transected and subsequently repaired.^29^ A rodent model of chronic nerve constriction found enhanced thalamic-S1 FC during the development of neuropathic pain,^73^ and stronger thalamic-S1 rsFC compared with HCs has been reported in other chronic pain conditions^74–76^ including upper extremity neuropathic pain, where stronger thalamic-S1 FC was associated with lower tactile acuity and weaker FC was linked with higher pain scores.^77^ Conversely, diabetic-neuropathy patients show reduced thalamic-S1 FC, particularly those with mechanical hyperalgesia.^38^ Interpreting the directionality of plasticity in thalamocortical connections is challenging when assessed with rs-fMRI, particularly in conditions that have heterogeneous (i.e. positive and negative) sensory and pain profiles.^15^ Contrary to our predictions, we found no differences in thalamocortical FC between patients pre-operatively and HCs. The heterogeneous pain and symptom scores in the present patient cohort may correspond with different compensatory or maladaptive changes throughout the somatosensory pathway. At the level of the peripheral nerve, multiple pathophysiological mechanisms likely interact, including mechanical forces, ischaemic compression, inflammation, oedema, and hypoxic injury,^1,78^ resulting in progressive axonal loss and deafferentation that affects both large (Aβ) and small (Aδ and C) sensory fibres.^3^ In the brain, S1 and M1 cortical thickness dissociates carpal tunnel syndrome patients with primarily paraesthesia versus pain symptoms.^79^ The amount of afferent drive from the hand in the present patient group likely differed depending on their symptoms and degree of nerve impairment, and the different symptom subtypes in carpal tunnel syndrome may require stratification when comparing with HCs to detect specific FC abnormalities.

Unlike our previous study in healthy young adults,^44^ higher TSP responses were not correlated with increased thalamic-S1 BA3a FC in either patients or HCs. Enhanced pain facilitation^80^ and TSP has been reported in carpal tunnel syndrome.^13^ These behaviours are considered experimental characteristics of central sensitization, and increased TSP is frequently (thought not always) reported in chronic pain conditions with ‘centralized’ pain.^81^ Contrary to our hypothesis, we found significantly less thermal TSP in both pre- and post-op patients compared with HCs. Significantly lower wind-up ratio (a measure similar to TSP) was also found in carpal tunnel syndrome versus HCs.^15^ While the area of the forearm where our TSP paradigm was performed is innervated by the lateral and medial antebrachial cutaneous nerve, some patients reported pain and paraesthesia symptoms extending up their volar forearm, which may have influenced TSP responses. Indeed, the patient group in this study had clinical characteristics of central sensitization, with 19 indicating proximal symptom spread, and seven showing extra-median symptom patterns.

Clinically, the carpal tunnel syndrome patients mostly had improved or resolution of pain and other sensory symptoms. However, there may be incomplete healing of the median nerve in line with Kennedy *et al*.^82^ The heterogeneity in sensory thresholds, extra-median versus median symptom distribution, and average pain and symptom scores likely reflects different carpal tunnel syndrome ‘subtypes’. Current clinical guidelines recommend that patients with ‘non-classical’ symptoms (i.e. extra-median and proximal spread) undergo painful electrophysiological testing to rule out involvement of other peripheral nerves.^83^ These tests are associated with increased costs and delays to surgery,^84^ which is linked with worse outcomes.^85^ Establishing the role of central plasticity and sensitization in carpal tunnel syndrome, particularly in non-classical symptoms, could reduce diagnostic uncertainty in these cases and perhaps even contribute to stratification of symptom subtypes for more personalized treatments.^1^ Characterizing altered cortical functioning in carpal tunnel syndrome could inform physiotherapy designed to target neuroplasticity, such as sensory re-learning, which has variable effectiveness.^86–88^ We recently demonstrated that abnormal rsFC of a node of the descending anti-nociceptive system, the subgenual anterior cingulate cortex (sgACC), was influenced by sex,^89^ as well as treatment-related plasticity in carpel tunnel syndrome.^90^ Thus, in addition to an impaired descending pain modulation system in chronic pain,^91^ the present study findings of altered thalamic-S1 and S1-to-whole brain FC help provide a more complete picture of the CNS aberrations in carpal tunnel syndrome.

There are limitations to our study, namely a small post-op patient sample size and insufficient healthy control sensory threshold data to enable us to definitively calculate sensory gain or loss in carpal tunnel syndrome patients relative to normal.^92^ As demonstrated by Kennedy *et al*.,^15^ healthy threshold data collected at the more common site of the thenar eminence are not suitable as a reference for finger thresholds. Therefore future studies of similar hand conditions could contribute to a shared database of QST measures performed on the fingers. Our sex-disaggregated analyses revealed that sex may influence carpal tunnel related-abnormalities in S1 FC, as it does in the descending anti-nociceptive system,^90^ however our sample of men may have been too small to detect the abnormally low S1-right somatosensory association cortex FC seen in the women only and main analyses (Supplementary Table 3). Finally, we note that three of our 21 patients were left-handed. Handedness is mostly a factor of importance in the motor and language systems and likely less so in studies of sensory/pain systems, although there is some evidence that handedness may influence resting-state FC in the somatosensory network of healthy children and young adults.^93^ Future studies with larger sample sizes should explore the effects of handedness and unilateral versus bilateral carpal tunnel syndrome symptoms on somatosensory FC.

In conclusion, we provide novel evidence that carpal tunnel syndrome is associated with altered FC in cortical areas associated with somatosensory and pain processing, and that surgical treatment can normalize some of these connectivities. Our findings of neuropathy- and treatment-related brain plasticity provide a window into potential mechanisms underlying the resolution of symptoms, and possible targeted treatments to complement hand surgery or provide relief for those with poor post-surgical outcomes.

## Supplementary Material

fcac237_Supplementary_Data

## Abbreviations

HCs =: healthy controls
post-op =: postoperative
pre-op =: preoperative
QST =: quantitative sensory testing
rsFC =: resting-state functional connectivity
rs-fMRI =: resting-state functional MRI
S1 =: primary somatosensory cortex
sgACC =: subgenual anterior cingulate cortex
TSP =: temporal summation of pain
