# Supplementary material for: Carpal tunnel surgery dampens thalamocortical and normalizes corticocortical functional connectivity
Source: Brain Commun. 2022 Sep 22;4(5):fcac237. doi: 10.1093/braincomms/fcac237 (PMC9556937; doi:10.1093/braincomms/fcac237)
Supplement: fcac237_Supplementary_Data [file fcac237_supplementary_data.pdf]

Carpal tunnel surgery dampens thalamocortical and normalizes corticocortical functional connectivity

Natalie R Osborne,[1,2] Dimitri J Anastakis,[1,2,3,4] Junseok Andrew Kim,[1,2] Rima El-Sayed,[1,2] Joshua C Cheng,[1,2] Anton Rogachov,[1,2] Kasey S Hemington,[1,2] Rachael L Bosma,[1] Camille Fauchon,[1] and Karen D Davis[1,2,3,4]

Supplementary Material Part 1: Patient Recruitment and Quantitative sensory testing

Methods

Dr. Anastakis and his team confirmed a diagnosis of carpal tunnel syndrome through clinical examination of each patient. Their assessment involved a thorough history of symptomology and clinical tests including Tinel's Sign,[1] Phalen's Test, Pressure Provocation Test[2] and two-point discrimination tests as well as assessments of grip strength, key pinch, muscle atrophy and abductor pollicis brevis tone.[3] In cases with diagnostic uncertainty nerve conduction studies were completed, but electrodiagnostic tests were not required for inclusion in the study because carpal tunnel syndrome is a clinical diagnosis.[4] Supplemental Table 1 provides a breakdown of each individual patient's age, sex, handedness, unilateral or bilateral CTS diagnosis, which hand(s) was operated on, and whether they had electrodiagnostic tests performed.

The QST session took place after neuroimaging data were acquired in a temperature-controlled testing room. The sensory detection thresholds were determined at two test sites on each hand: the volar aspect of the distal phalanx (fingertip) for D2 (index finger) and D5 (pinky finger). The testing order (e.g., right vs. left hand) was alternated subject to subject but not within subject for the HCs. For the patients, test site order was based on their symptoms; the least affected site (i.e., pinky finger of their least affected hand) was tested first, and the most affected site (i.e., index finger of their most symptomatic hand) was tested last.

Participants closed their eyes and rested their hand, palm up, on a supportive surface while mechanical and vibration detection thresholds were acquired. Mechanical detection thresholds were determined using a standardized set of von Frey filaments (OptiHair2 Marstock Nervtest, Germany), which contains 12 logarithmically spaced calibrated filaments that deliver forces from 0.25–512 mN with a contact surface of 0.4 mm. Probes were applied in an ascending series until the participant verbally indicated they had felt the probe touch their fingertip. This was repeated two more times, and the logarithmic mean of all three trials was calculated. We determined vibration detection thresholds using the method of limits with a hand-held Bio-Thesiometer (Bio-Medical Instrument Company, USA). The device's 12-mm probe was placed on the fingertip and the stimulus amplitude (voltage) was gradually increased until the participant indicated that they perceived a vibration sensation. This was repeated two more times, and the average of the three trials was calculated.

Thermal thresholds were determined based on the method of limits using a computer-controlled Peltier device (TSA-II–Neurosensory Analyzer; Medoc Ltd, Israel) with a 15 X 15 mm thermode secured to the fingertip as the participant's hand rested palm up on a supportive surface. The participant's other hand rested on a mouse which they were instructed to click to indicate the moment they perceived the sensation being assessed, at which point the thermode temperature would return to baseline (32˚C) in preparation for the next trial. There were three consecutive trials for each threshold. The method of limits was used in the following order: warm detection threshold (WDT), heat pain threshold (HPT), cool detection threshold (CDT) and cold pain threshold (CPT). Temperature was ramped from baseline at a rate of 0.5˚C/s. Cutoff temperatures were set to 0˚C for CDT and CPT, and 50˚C for WDT and HPT. Thermal thresholds were calculated by averaging the last two trials.

We then determined the temperature that elicited a verbal pain rating of 50/100 (where 0 is no pain and 100 is the worst pain imaginable). To do this, participants underwent a familiarization paradigm, where a 30 x 30 mm thermode is strapped to the left volar forearm, 15 cm from the wrist. Different thermal intensities are delivered and the participant is asked to verbally rate the pain of each stimulus (0-100). The thermal stimuli increase from baseline with a ramp rate of 2°C/s and maintain their target temperature for 6s, with rest periods in between each stimuli. They are delivered in the following order; 44°C, 45°C, 43°C, 46°C, 42°C, and if the participant did not rate any of the previous stimuli greater than 75/100, a final stimulus of 47°C is delivered. If one of the thermal stimuli from this familiarization protocol elicited a pain rating of 50-60/100, that temperature is taken as the participants Pain50. If not, a stimulus at a temperature that was 1˚C above their pain threshold was delivered at a ramp rate of 1˚C/s and maintained for 7 seconds. The participants were asked to provide a verbal pain rating (0-100), and then the temperature returned to baseline (32 ˚C). This process was repeated, adjusting the temperature up or down by 0.3 ˚C each trial, until a temperature that elicited a pain rating between 50-60 was achieved, referred to as the participant's "Pain50".

The Pain50 temperature was subsequently used in the temporal summation of pain (TSP) paradigm, a behavioural measure thought to reflect central sensitization, described in the Methods section of the main manuscript. The location of the familiarization protocol on the volar forearm was chosen for two reasons. The first was to keep this measurement consistent across our previously established healthy control database as well as several other patient databases that use the same protocol to determine pain50 as well as the same thermal TSP protocol. This enables us to compare results from these two quantitative sensory testing measures between our patients and controls and allows for future studies to investigate these measures across various chronic pain groups. Secondly, the location of the thermal familiarization and TSP protocols (15 cm from wrist) was purposefully kept outside the site of primary injury (i.e., median nerve innervated territory) because the intention of the TSP paradigm was to assess central sensitization in this patient group. Behavioural signs of central sensitization in patients with carpal tunnel syndrome have previously been reported as secondary hyperalgesia at distal sites on the affected arm, unaffected arm, or distant body sites including the leg and foot[7-9], as well as enhanced TSP[10]. We wanted to avoid any primary hyperalgesia at the "injury site" influencing the temperature used to determine pain50, and subsequent performance in the TSP paradigm.

Results

Pre and post-operative sensory threshold data collected from the index finger of patient’s most affected hand, along with HC data (n=10) and Pain50 data for all participants are presented in the Results section of the main manuscript as well as Figure 2. Here, pre and post-operative sensory threshold data across all testing sites (index and pinky fingers of both hands) are presented in Supplemental Table 2.

References

1. Kuschner SH, Ebramzadeh E, Johnson D, Brien WW, Sherman R. Tinel's sign and Phalen's test in carpal tunnel syndrome. *Orthopedics*. Nov 1992;15(11):1297-302.
2. Williams TM, Mackinnon SE, Novak CB, McCabe S, Kelly L. Verification of the pressure provocative test in carpal tunnel syndrome. *Ann Plast Surg*. Jul 1992;29(1):8-11. doi:10.1097/00000637-199207000-00003
3. MacDermid JC, Wessel J. Clinical diagnosis of carpal tunnel syndrome: a systematic review. *J Hand Ther*. Apr-Jun 2004;17(2):309-19. doi:10.1197/j.jht.2004.02.015
4. Mooar PA, Doherty WJ, Murray JN, Pezold R, Sevarino KS. Management of Carpal Tunnel Syndrome. *J Am Acad Orthop Surg*. Mar 15 2018;26(6):e128-e130. doi:10.5435/JAAOS-D-17-00451

Supplemental Table 1: Individual characteristics of patients with CTS

Patient	Age	Sex	Handedness	CTS Diagnosis	EDX confirmed	Operated Hand	2nd Operated Hand
1	60	M	Right	Bilateral (L>R)	n/a	Left	Right
2	42	F	Right	Bilateral (L>R)	Yes	Left	Right
3	37	F	Right	Bilateral (R>L)	Yes	Right	Left
4	49	F	Right	Bilateral (R>L)	Yes	Right	
5	37	F	Left	Bilateral (L>R)	Yes	Left	Right
6	66	F	Left	Bilateral (L>R)	Yes	Left	
7	48	F	Right	Unilateral (R)	Yes	Right	
8	54	F	Right	Bilateral (L>R)	Yes	Left	Right
9	33	F	Right	Bilateral (R>L)	Yes	Right	Left
10	35	M	Right	Bilateral (R>L)	Yes	Right	Left
11	34	F	Left	Bilateral (L>R)	n/a	Left	Right
12	40	M	Ambidextrous	Bilateral (R>L)	Yes	Right	
13	68	F	Right	Unilateral (R)	n/a	Right	
14	40	M	Right	Bilateral (R>L)	Yes	Right	Left
15	43	M	Right	Bilateral (R>L)	Yes	Right	
16	62	F	Right	Bilateral (R>L)	n/a	Right	
17	60	F	Right	Bilateral (L>R)	Yes	Left	
18	63	F	Right	Bilateral (R>L)	Yes	Right	
19	60	F	Right	Bilateral (R>L)	n/a	Right	Left
20	61	M	Right	Bilateral (R>L)	n/a	Right	Left
21	60	M	Right	Bilateral (R>L)	n/a	Right	
22	57	F	Right	Bilateral (R>L)	Yes	Right	Left
23	30	F	Right	Bilateral (R>L)	Yes	Left	
24	47	F	Right	Bilateral (R>L)	n/a	Right	Left
25	49	F	Right	Bilateral (R>L)	n/a	Right	Left

Demographic and clinical characteristics of patients with CTS who had carpal tunnel release surgery performed on either one or both hands. F=female, M=male, R>L = right hand more severe than left, L>R = left hand more severe than right, EDX confirmed = electrodiagnostic tests confirmed CTS diagnosis, n/a = electrodiagnostic tests were not available for this patient.

Supplemental Table 2: Sensory thresholds in patients with carpal tunnel syndrome

	**Pre-Operative**				**Post-Operative**			
**Hand**	**Most Affected**		**Less Affected[a]**		**1st Operated**		**2nd or non-operated[b]**	
**Test Site**	**D2**	**D5**	**D2**	**D5**	**D2**	**D5**	**D2**	**D5**
**MDT, mN**<br>*Pre n=22;*<br>*Post n=14*	**6.6 ± 21.3**	3.0 ± 8.4	2.0 ± 4.2	1.7 ± 4.2	**1.0 ± 1.0***	0.8 ± 0.9	1.3 ± 1.6	0.5 ± 0.1
**VDT, V**<br>*Pre n=20;*<br>*Post n=14*	**4.7 ± 3.0**	3.9 ± 2.3	4.0 ± 2.0	4.6 ± 4.3	**3.0 ± 1.1***	3.4 ± 1.5	3.4 ± 1.6	2.9 ± 1.4
**WDT, °C**<br>*Pre n=15;*<br>*Post n=14*	**36.8 ± 2.5**	38.0 ± 3.9	36.8 ± 3.1	37.4 ± 4.3	**36.6 ± 3.7**	37.1 ± 3.2	36.0 ± 1.6	37.4 ± 3.6
**HPT, °C**<br>*Pre n=15;*<br>*Post n=14*	**45.8 ± 4.0**	45.7 ± 3.1	45.0 ± 4.2	45.8 ± 3.9	**44.1 ± 4.4***	44.6 ± 2.9	44.1 ± 4.2	44.7 ± 3.5
**CDT, °C**<br>*Pre n=14;*<br>*Post n=13*	**24.1 ± 7.3**	17.7 ± 8.4	24.9 ± 8.0	21.7 ± 9.3	**27.3 ± 4.7***	23.9 ± 7.4	27.7 ± 2.9	26.1 ± 9.0
**CPT, °C**<br>*Pre n=14;*<br>*Post n=13*	**9.0 ± 11.2**	7.5 ± 6.4	12.1 ± 10.1	8.8 ± 8.3	**13.3 ± 10.5**	13.1 ± 7.7	13.6 ± 10.9	12.9 ± 9.8

Group data are displayed as mean ± standard deviation.

* $P < 0.05$. One-tailed paired t-tests compared pre-operative thresholds on the most affected digit (D2, index finger) of the most affected hand with post-operative thresholds on that same digit (bolded data). Exact *P* values are reported in the Results section of the main manuscript.

[a]Two patients had unilateral CTS affecting the right hand only (therefore, their left hand would be considered "unaffected"), while the remaining patients had bilateral CTS

[b]Seven of the patients who returned for their post-op study visit had carpal tunnel release surgery on both of their hands (surgeries were performed one at a time, and post-op visits took place at least three months after the second surgery), and nine patients had surgery on just one hand

D2 = digit 2 (index finger), D5 = digit 4 (pinky finger), MDT = mechanical detection threshold, VDT = vibration detection threshold, WDT = warm detection threshold, HPT = heat pain threshold, CDT = cool detection threshold, CPT = cold pain threshold

Supplementary Material Part 2: Additional Neuroimaging Analyses

Dynamic FC between the thalamus and S1

Methods

We determined the dynamic FC between our thalamus and S1 BA3a seeds using the dynamic conditional correlation method used previously.[1-3] Each participants' time course was pre-whitened using an autoregressive and moving average (ARMA) (1,1) model. A generalized autoregressive conditional heteroscedastic (GARCH) model was then applied to estimate the conditional standard deviation (SD) over time, which was used to standardize the residuals of the time series. We used the dynamic conditional correlation to calculate the time-varying correlation between the time series with an exponentially weighted moving average derived from the data using maximum likelihood.[4] We then calculated the SD of each dynamic conditional correlation across the time series and used this SD as the summary metric for dynamic FC.[5] Higher SD values indicate greater fluctuations in FC strength between two brain regions over time. We calculated the dynamic conditional correlation for each pre-op patient and HC between our left thalamus and left S1 BA3a seeds. This provided us with a SD value representing the dynamic FC between these two seeds, which we compared between pre-operative and post-operative patients with carpal tunnel syndrome and HCs in GraphPad Prism 7 using two independent-samples t-tests.

Results

There was no significant difference in dFC between the thalamus and S1 BA3a seed between pre-operative patients and HCs (two-tailed two sample t-test, P=0.4519), or between post-operative patients and HCs (two-tailed unpaired t-test, P=0.8830). See Supplemental Figure 1.

Sex-disaggregated FC analyses in patients with carpal tunnel syndrome and HCs

Carpal tunnel syndrome is more prevalent in women,[6,7] with some evidence that women report worse sensory symptoms than men with the same level of nerve impairment.[8,9] While we previously demonstrated resting state FC abnormalities in the descending antinociceptive system that are influenced by sex,[10] we did not have specific hypotheses about whether sex would influence S1 and thalamic FC in these patients. The smaller number of men (n=7) compared to women (n=18) in the patient sample precluded a direct statistical comparison between sex in this study. Therefore, to investigate a role sex may have on the abnormality in S1-somatosensory association cortex FC revealed in Analysis 1a, we performed sex-disaggregated static FC analyses of the S1 hand area to whole brain. Although Analysis 2a did not reveal any abnormalities in thalamocortical FC in pre-op carpal tunnel patients, we included a sex-disaggregated version of this analysis to investigate potential influences of sex on thalamic FC.

Methods

To perform the sex-disaggregated S1 hand area seed-to-whole brain FC analyses, we used the first-level GLM analyses created in Analysis 1a and entered them into two separate second-level FEAT analyses (two-group difference model with FLAME 1+2), one comparing women with carpal tunnel syndrome to healthy women (n=32), and the other comparing men with carpal tunnel syndrome to healthy men (n=14). We repeated this same procedure for the thalamic to somatosensory mask analyses, using first-level GLMs created in Analysis 2a. A cluster-based statistical threshold of $Z > 2.3$ and $P < 0.05$ with a Family Wise Error (FWE) correction was used for all FC analyses.

Results

S1 to whole brain FC in pre-operative patients vs. HCs

We conducted sex-disaggregated analyses to investigate the potential influence of sex on our finding of abnormally low left S1 hand area to right somatosensory association cortex FC in carpal tunnel patients pre-operatively. The main finding of reduced S1-right supramarginal (BA 40) FC was identified in women with carpal tunnel syndrome compared to healthy women (n=36, P=0.001), but not in men with this condition compared to healthy men (n=14). Additionally, women with carpal tunnel syndrome had stronger S1 FC with a cluster including the right angular gyrus (BA 39), fusiform gyrus (BA 37) and lateral occipital cortex compared to healthy women (P=0.0289), while men with carpal tunnel syndrome had stronger FC with bilateral primary visual areas (BA 17) than healthy men (P=0.03). See Supplemental Table 3.

Thalamocortical FC in pre-operative patients vs. HCs

There were no differences between women with carpal tunnel syndrome and healthy women, nor men with carpal tunnel syndrome and healthy men when it came to thalamic FC with the somatosensory system mask.

References

1. Bosma RL, Cheng JC, Rogachov A, et al. Brain Dynamics and Temporal Summation of Pain Predicts Neuropathic Pain Relief from Ketamine Infusion. *Anesthesiology*. Nov 2018;129(5):1015-1024. doi:10.1097/ALN.0000000000002417
2. Cheng JC, Rogachov A, Hemington KS, et al. Multivariate machine learning distinguishes cross-network dynamic functional connectivity patterns in state and trait neuropathic pain. *Pain*. Sep 2018;159(9):1764-1776. doi:10.1097/j.pain.0000000000001264
3. Rogachov A, Bhatia A, Cheng JC, et al. Plasticity in the dynamic pain connectome associated with ketamine-induced neuropathic pain relief. *Pain*. Jul 2019;160(7):1670-1679. doi:10.1097/j.pain.0000000000001545
4. Lindquist MA, Xu Y, Nebel MB, Caffo BS. Evaluating dynamic bivariate correlations in resting-state fMRI: a comparison study and a new approach. *Neuroimage*. Nov 1 2014;101:531-46. doi:10.1016/j.neuroimage.2014.06.052
5. Choe AS, Nebel MB, Barber AD, et al. Comparing test-retest reliability of dynamic functional connectivity methods. *Neuroimage*. Sep 2017;158:155-175. doi:10.1016/j.neuroimage.2017.07.005

6. Aroori S, Spence RA. Carpal tunnel syndrome. *Ulster Med J*. Jan 2008;77(1):6-17.
7. MacDermid JC, Wessel J. Clinical diagnosis of carpal tunnel syndrome: a systematic review. *J Hand Ther*. Apr-Jun 2004;17(2):309-19. doi:10.1197/j.jht.2004.02.015
8. Mondelli M, Aprile I, Ballerini M, et al. Sex differences in carpal tunnel syndrome: comparison of surgical and non-surgical populations. *Eur J Neurol*. Dec 2005;12(12):976-83. doi:10.1111/j.1468-1331.2005.01099.x
9. Ferry S, Hannaford P, Warskyj M, Lewis M, Croft P. Carpal tunnel syndrome: a nested case-control study of risk factors in women. *Am J Epidemiol*. Mar 15 2000;151(6):566-74. doi:10.1093/oxfordjournals.aje.a010244
10. Osborne NR, Anastakis DJ, Kim JA, et al. Sex-Specific Abnormalities and Treatment-Related Plasticity of Subgenual Anterior Cingulate Cortex Functional Connectivity in Chronic Pain. Original Research. *Frontiers in Pain Research*. 2021-July-12 2021;2(9)doi:10.3389/fpain.2021.673538

Supplemental Table 3: Findings from sex disaggregated neuroimaging analyses

S1 hand area to whole brain FC, CTS vs HC Women only (*n*=36)

**Contrast**	***Z***	***P***	**MNI Coordinates**			**Brain Regions**
			**x**	**y**	**z**	
CTS > HC	3.61	0.0289	60	-66	16	BA 39, Angular Gyrus<br>BA 37, Fusiform Gyrus<br>Lateral Occipital Cortex
CTS < HC	4.45	0.00123	60	-32	46	BA 40, Somatosensory Association Cortex<br>Inferior Parietal Lobule (Supramarginal & Angular Gyri)

S1 hand area to whole brain FC, CTS vs HC Men only (*n*=14)

CTS > HC	4.27	0.0299	6	-98	0	BA 17, Bilateral Primary Visual Area (Supra & Intracalcarine Cortex, Occipital Pole)

Peak MNI coordinates, Z, and *P* values are reported for significant clusters found in the S1 (hand area) seed-to-voxel whole brain functional connectivity analyses comparing women with carpal tunnel syndrome (CTS) before surgery to HC women (*n*=36), and men with CTS before surgery to HC men (*n*=14). Brain regions are provided using FSL's Harvard-Oxford Cortical Atlas & Talairach Daemon Label tools. Thresholded at *P* <0.05 (FWE-corrected for multiple comparisons).

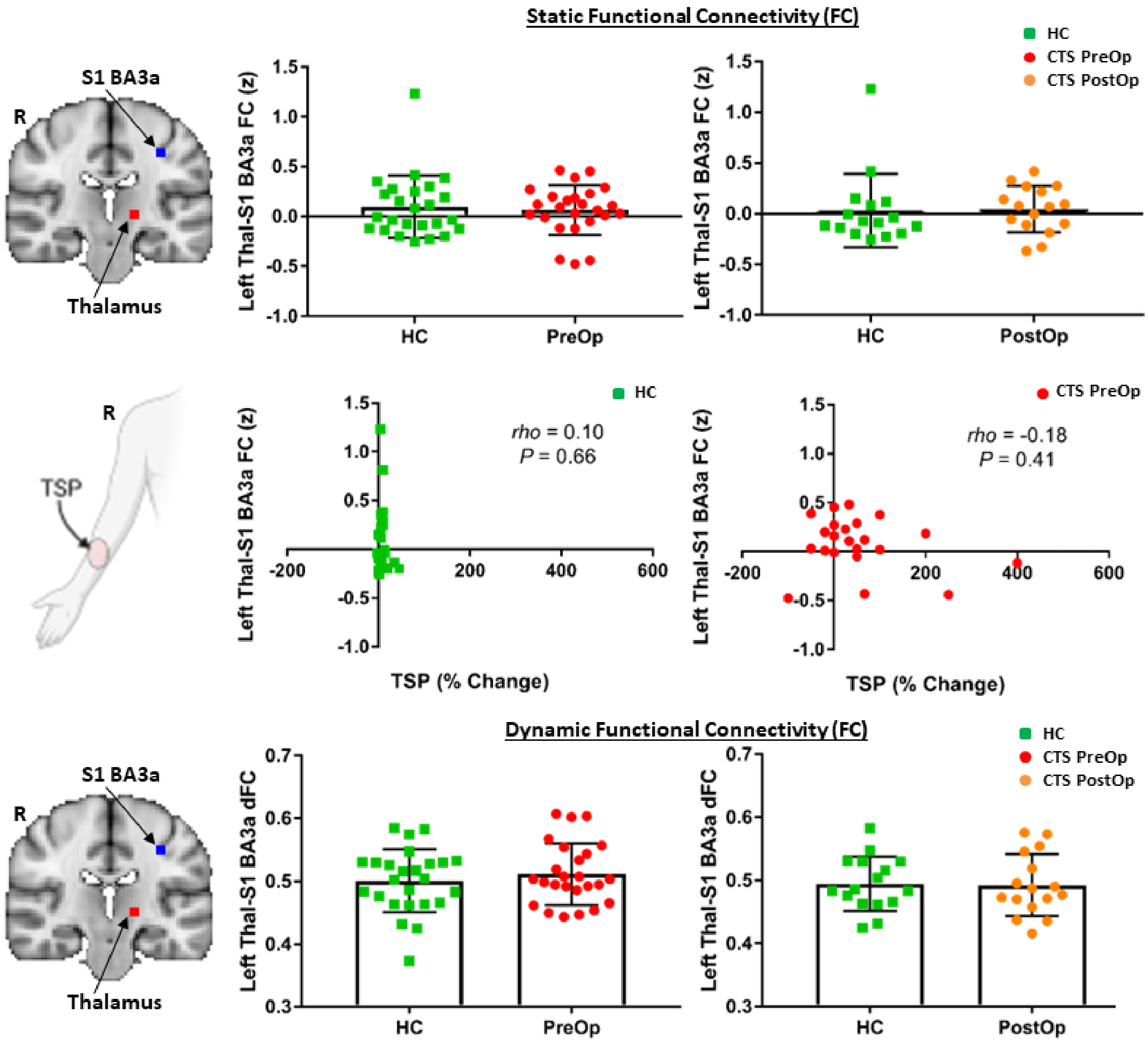

Supplemental Figure 1 Normal thalamus to S1 BA3a FC in carpal tunnel syndrome. Our seed-to-seed analyses of static FC between the left thalamus and left S1 BA3a area revealed no differences between pre-op patients with carpal tunnel syndrome (*n*=25) and HCs (Mann-Whitney two-tailed test, *U*=291, *P*=0.6862) or post-op patients (*n*=16) and HCs (Mann-Whitney two-tailed test, *U*=100, *P*=0.3045). Furthermore, pre-operative FC between these two regions was not correlated with pre-operative TSP responses in either patients (Spearman's *rho*=-0.1845, *P*=0.411) or controls (Spearman's *rho*=0.1008, *P*=0.664). There was no significant difference in dynamic FC between the left thalamus and S1 BA3a seed between pre-op patients and HCs (unpaired two-tailed t-test, t(48)=0.7584, *P*=0.4519) or post-op patients and HCs (unpaired two-tailed t-test, t(30)=0.1485, *P*=0.8830). Significance was set at *P*<0.05, error bars represent SD. Created with BioRender.com. S1 = Primary somatosensory cortex, FC = functional connectivity, BA = Broadman Area, TSP = temporal summation of pain, PreOp = Pre-operative, PostOp = Post-operative, HC = healthy control, R= right.

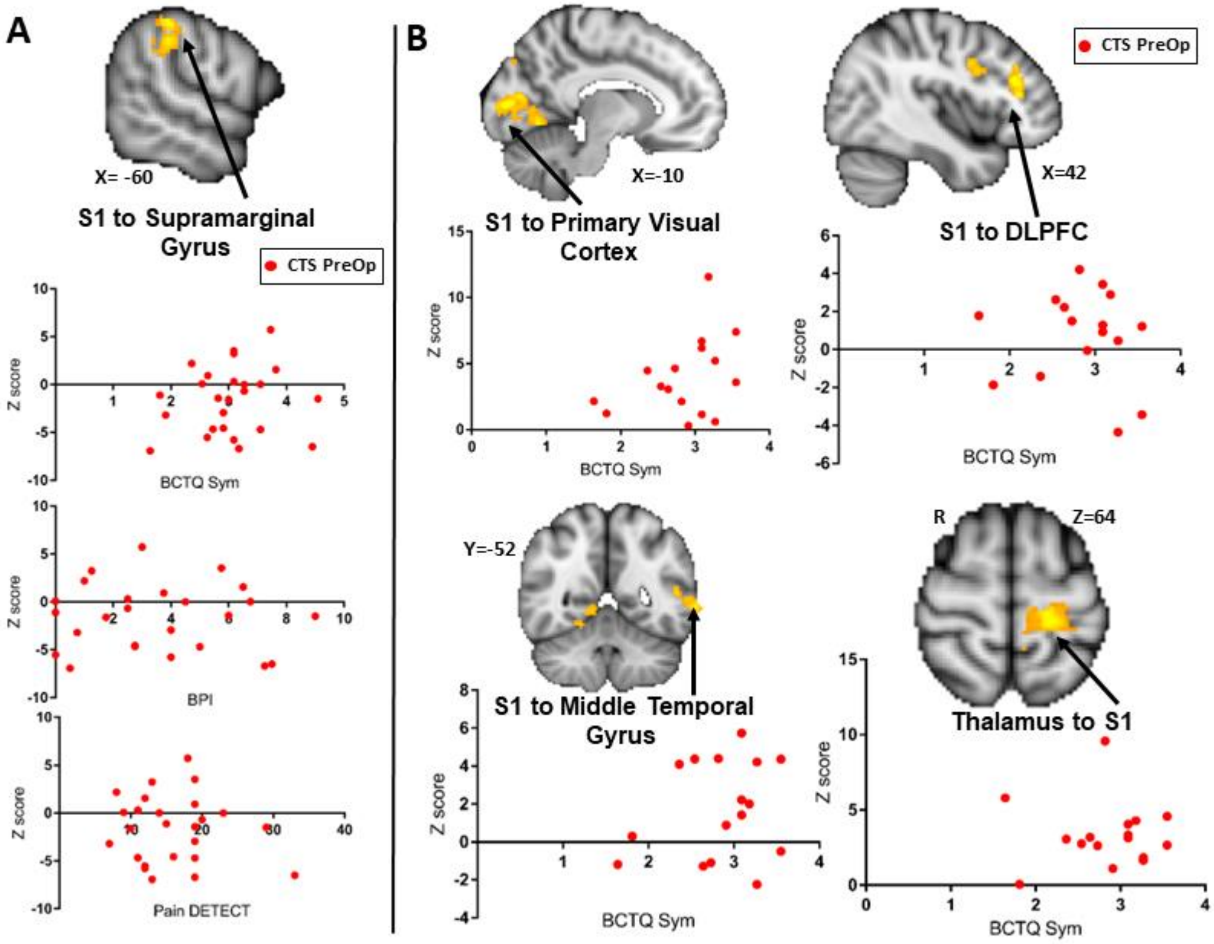

Supplemental Figure 2 No Correlations between FC and clinical measures. In Analysis 3, pre-operative patients' first-level S1 and thalamus FC analyses were entered into second-level regression analyses in FEAT (single-group average with additional covariate), with the statistically significant clusters from the group difference used as a mask, and the demeaned pre-op clinical scores entered as a covariate. We did not find significant correlations between pre-operative patients' clinical scores and their FC. Because FEAT does not provide statistical values for results that do not meet the threshold of significance, we performed the correlations in GraphPad Prism 7.0 and graphed the results here for visualization purposes. Specifically, we ran correlations between pre-operative patients' (n=25) mean Z statistic from a 2mm spherical seed centered around peak coordinates of the significant cluster being investigated (y-axis) and their pre-op BCTQ, BPI or pain DETECT scores (x-axis). **(A)** Regression analyses in FEAT (single group average with additional covariate) masked with a significant cluster in the supramarginal gyrus **(**revealed to have abnormally low FC compared to HCs in Analysis 1a) did not find significant relationships between pre-operative S1 FC with this region and pre-op BCTQ symptom, BPI, and painDETECT scores. This was confirmed with subsequent correlation analyses performed in GraphPad between S1-supramarginal gyrus FC and BCTQ symptom scores (Pearson's r=0.1289, P=0.539), BPI scores (Pearson's r=-0.05608, P=0.790) and painDETECT scores (Pearson's r=-0.1188, P=0.572). **(B)** A regression analysis in FEAT masked with clusters in the primary visual cortex, DLPFC and middle

temporal gyrus (regions where S1 FC was attenuated following surgery, Analysis 1B) revealed no significant relationships between pre-operative patients' S1 FC with these regions and their pre-op BCTQ symptom scores. Subsequent analyses performed in GraphPad illustrate this lack of significant correlation between BCTQ symptom scores and FC between S1-V1 (Pearson's r=0.371, P=0.152), S1-DLPFC (Pearson's r=-0.09802, P=0.718) and S1-middle temporal gyrus (Pearson's r=0.2186, P=0.416). A similar analysis, masked with a cluster in the S1 and M1 (for which thalamic FC was attenuated post-op, Analysis 2B) did not find a relationship between thalamic FC with this region and pre-op BCTQ symptom scores. This is illustrated by a correlation in GraphPad between thalamic-S1/M1 FC and BCTQ symptom score (Spearman's *rho*=0.004431, P=0.989). FC = functional connectivity, S1 = primary somatosensory cortex, M1 = primary motor cortex, DLPFC = dorsolateral prefrontal cortex, HC = healthy controls, BCTQ = Boston Carpal Tunnel Questionnaire, BPI = Brief Pain Inventory, R= right, Post = Post-operative, Pre = Pre-operative.
